# A Show of Ewald's Law: I Horizontal Semicircular Canal Benign Paroxysmal Positional Vertigo

**DOI:** 10.3389/fneur.2021.632489

**Published:** 2021-02-03

**Authors:** Xueqing Zhang, Yanru Bai, Taisheng Chen, Wei Wang, Xi Han, Shanshan Li, Qiang Liu, Chao Wen

**Affiliations:** ^1^Department of Otorhinolaryngology Head and Neck Surgery, Tianjin First Central Hospital, Tianjin, China; ^2^Tianjin First Central Hospital, Institute of Otolaryngology of Tianjin, Tianjin, China; ^3^Key Laboratory of Auditory Speech and Balance Medicine, Department of Otorhinolaryngology Head and Neck Surgery, Tianjin First Central Hospital, Tianjin, China; ^4^Key Clinical Discipline of Tianjin (Otolaryngology), Tianjin, China; ^5^Otolaryngology Clinical Quality Control Centre, Tianjin, China; ^6^Academy of Medical Engineering and Translational Medicine, Tianjin University, Tianjin, China

**Keywords:** canalithiasis, horizontal semicircular canal, otolithic membrane, video nystagmography, Ewald's laws

## Abstract

**Objective:** To evaluate horizontal semicircular canal (HSC) effects according to Ewald's law and nystagmus characteristics of horizontal semicircular canal benign paroxysmal positional vertigo (HSC-BPPV) in the supine roll test.

**Methods:** Patients with HSC-BPPV (*n* = 72) and healthy subjects (*n* = 38) were enrolled. Latency, duration, and intensity of nystagmus elicited by supine roll test were recorded using video nystagmography.

**Results:** In patients with HSC-BPPV, horizontal nystagmus could be elicited by right/left head position (positional nystagmus) and during head-turning (head-turning nystagmus), and nystagmus direction was the same as that of head turning. Mean intensity values of head-turning nystagmus in HSC-BPPV patients were (44.70 ± 18.24)°/s and (44.65 ± 19.27)°/s on the affected and unaffected sides, respectively, which was not a significant difference (*p* = 0.980), while those for positional nystagmus were (40.81 ± 25.56)°/s and (17.69 ± 9.31)°/s (ratio, 2.59 ± 1.98:1), respectively, representing a significant difference (*p* < 0.0001). There was no positional nystagmus in 49 HSC-BPPV patients after repositioning treatment, nor in the 38 healthy subjects. No significant difference in head-turning nystagmus was detected in HSC-BPPV patients with or without repositioning.

**Conclusions:** The direction and intensity of nystagmus elicited by supine roll test in patients with HSC-BPPV, was broadly consistent with the physiological nystagmus associated with a same HSC with single factor stimulus. Our findings suggest that HSC-BPPV can be a show of Ewald's law in human body.

## Introduction

Benign paroxysmal positional vertigo (BPPV), the most common peripheral vestibular disorder, is characteristic by recurrent attacks of brief positional vertigo (dizziness) and nystagmus elicited by a change in head position relative to gravity (1). The horizontal semicircular canal (HSC) is the second most affected canal, with HSC-BPPV accounting for 5–30% of all BPPV cases (2). In patients with HSC-BPPV, direction-changing positional nystagmus—horizontal, not vertical—can be elicited by specific diagnostic positional maneuvers, such as the supine roll test. In 1824, Marie–Jean–Pierre Flourens first found the relationship of eye movement and canals through damage to the pigeons' semicircular canals caused changes in their behavior (3). Since then, J. Richard Ewald conducted more elaborate experiments in 1892 (4). He observed the intensity and direction of nystagmus by inserting a small tube into the semicircular canal of the pigeon and applying positive and negative pressure. Flourens' and Ewald's Laws play an important role in our understanding of the physiology of human semicircular canals and the diagnosis of vestibular disorders. Both were derived from animal experiments, while a functional model of a single semicircular canal has not been established in human body.

At present, the diagnosis of HSC-BPPV was mainly based on the visual observation of nystagmus, and its excitatory or inhibitory effects have not been quantified. Caloric test is a classical method for research into the function of the HSC, however, it is difficult to stimulate the unilateral horizontal semicircular canal with a single factor (5). In HSC-BPPV, rolling of the otoconia from the posterior arm of the HC toward the ampulla in the affected side due to gravity drives the endolymph to the ampulla, while the otoconia rolls to the canal from the ampulla, driving the endolymph away from the ampulla (6, 7). Therefore, HSC-BPPV could reflect the direction and intensity of the nystagmus elicited by equal excitation and inhibition on a single horizontal semicircular canal, and clarify the gradient of those effects, which will be helpful in understanding the functional status of single horizontal semicircular canals.

In this study, we recorded and analyzed the latency time, direction, intensity, and duration of nystagmus in patients with HSC-BPPV during the supine roll test using 2-dimensional video nystagmography (2-D VNG). Further, we discussed the internal relationship between HSC-BPPV and Ewald's law, providing a basis for deeper understanding of the physiological characteristics of the human horizontal semicircular canal.

## Materials and Methods

### Subjects

This was a prospective study involving assessment of 72 patients with vertigo, examined at the ENT Department of MY Hospital, Tianjin First Central Hospital between July 2018 and February 2019. Of 72 patients, 49 had accepted repositioning treatment and 23 had accepted other treatment. Healthy subjects (*n* = 38) were recruited as a control group. All subjects provided informed consent prior to their inclusion in the study. The study procedures have been approved by the Ethics Committee of the Tianjin First Central Hospital.

Inclusion Criteria:

1. Patients with a history of vertigo as a predominant symptom, or associated with other complaints, such as dizziness, vomiting, headache, hearing loss, etc.
2. Patients diagnosed with geotropic HSC-BPPV, according to the *Clinical practice guideline: benign paroxysmal positional vertigo* (8).

Exclusion Criteria:

1. Patients with apogeotropic HSC-BPPV, SSC-BPPV, PSC-BPPV, multiple-canal BPPV, cupulolithiasis, spontaneous, or other types of positional nystagmus.
2. Patients with neurological deficits, including hemiplegia, quadriplegia, and stroke (cerebrovascular accident).

### Methods

A detailed medical history, with a primary focus on the type of vertigo, and including the onset of symptoms, as well as their severity, duration, and associated factors, was obtained. Induction of nystagmus and corresponding parameters were observed and recorded using 2-D VNG (France Synapsys) during supine roll and Dix-Hallpike tests.

The supine roll test was performed to diagnose HSC-BPPV, and consisted of turning the head from the supine to either lateral position, when the patient was lying down in a supine position, with the head maintained at a 30 degree upward angle. The remaining steps of the exercise were the same as those for the supine roll test in *Clinical Practice Guideline* (9). The direction, latency time, duration time, and intensity of nystagmus were recorded in the supine position, left head position, and right head position, as well as during the process of head-turning. Latency time was the period from the end of a head turn (right/left head position) to the onset of continuous horizontal nystagmus. Duration was the period of continuous horizontal nystagmus from the onset to the end. The peak slow-phase velocity within 10 s from the onset of nystagmus was recorded as the intensity of nystagmus. Nystagmus direction and intensity were recorded in the supine and left/right head-turning positions during the supine roll test. Dix-Hallpike test was used as a preliminary method to analyze the characteristics of nystagmus and identify PSC-BPPV and multiple-canal BPPV, and its relevant data were not included in this data analysis. Based on the evidence on cupulolithiasis or canalithiasis in BPPV, multiple countries have established similar guidelines for the diagnosis and treatment of BPPV, with the canalith repositioning procedure (CRP) the primary treatment approach used (10).

### Analysis

The parameters of horizontal nystagmus elicited by the supine roll test were compared within and between groups. IBM SPSS Statistics 22 (IBM SPSS, Turkey) was used for statistical analyses and GraphPad Prism 5 (GraphPad, San Diego, CA, USA) and R scripts were used to generate figures.

## Results

### General Demographic Characteristics of Subjects

Patients with HSC-BPPV (18 men and 54 women) ranged in age from 21 to 81 years (mean 53.3 years). The control group comprised 38 healthy subjects (9 men and 29 women; mean age 52.8 years; age range 20–70 years). Demographic data for BPPV and control groups are summarized in Table 1. There were no significant differences in age or sex ratio between the two groups (*p* > 0.05). The supine roll test was performed on both sides for all patients with HSC-BPPV and controls, among which 49 cases with HSC-BPPV underwent successful repositioning using the barbecue maneuver.

**Table 1:** Demographic features of subjects in the HSC-BPPV and control groups.

**Group feature**	**Control**	**HSC-BPPV**	
		**Before reposition**	**After reposition**
Number	38	72	49
Age (years)^*^	52.7 ± 15.5	53.3 ± 14.8	53.6 ± 14.4
Sex (M:F)^*^	9:29	18:54	13:36

*HSC-BPPV, horizontal semicircular canal benign paroxysmal positional vertigo; M, male; F, female*;

* *p > 0.05*.

### General Characteristics of Nystagmus in Patients With HSC-BPPV on Supine Roll Test

**Figure 1:**
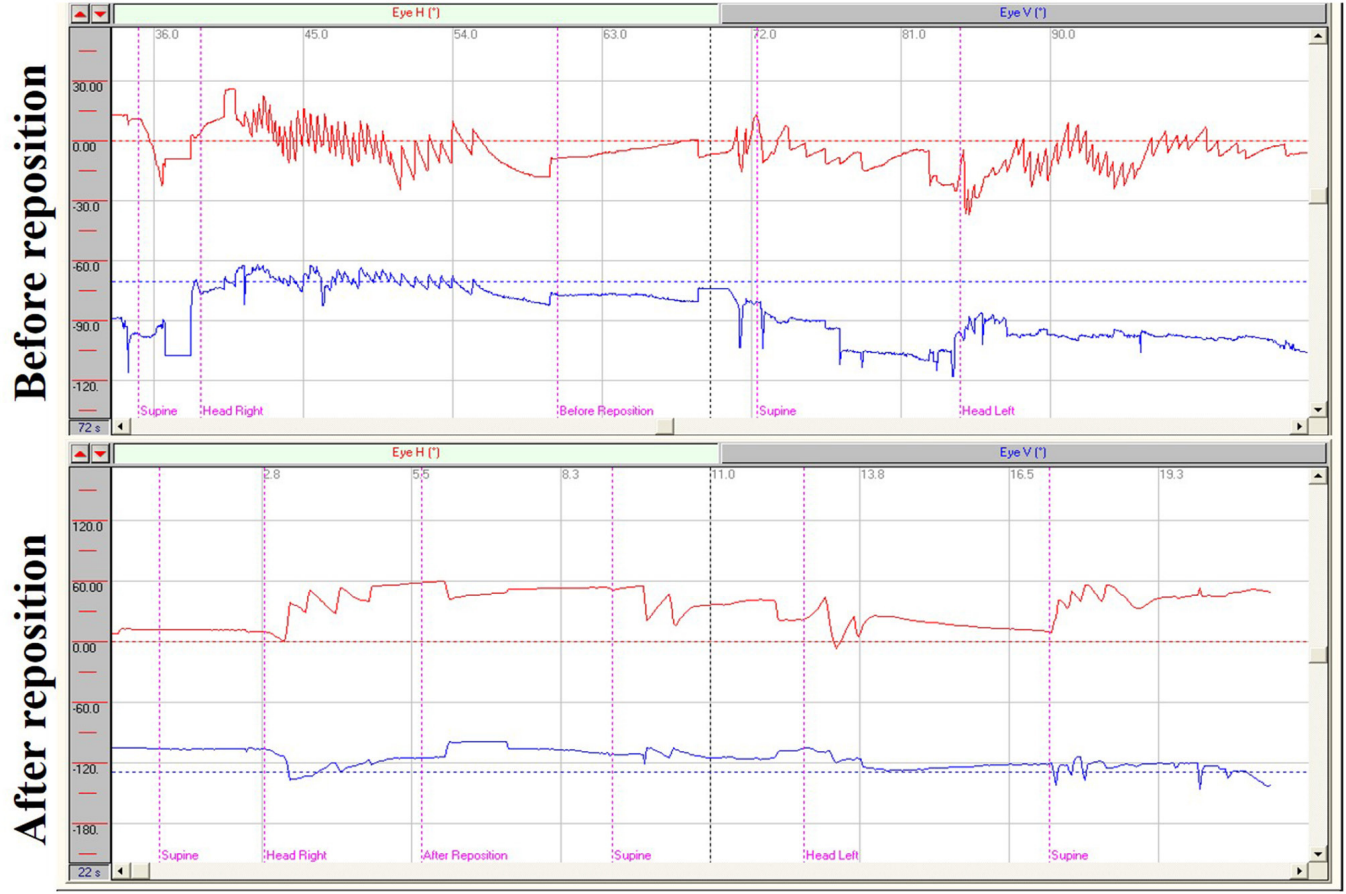
Nystagmus measurements from supine roll tests of a patient with right HSC-BPPV before and after repositioning. **Before reposition**: Right horizontal positional nystagmus (72.5°/s), accompanied by weak vertical upward nystagmus, was elicited when the patient was moved from supine to head-right position and left horizontal positional nystagmus (35.7°/s) was elicited when the patient was moved from the supine to head-left position. Head-turning nystagmus was elicited in the same direction when the patient was moved from the head-right to supine position as from the supine to the head-left position, and vice versa. **After reposition:** Only head-turning nystagmus was detected on supine roll test of the patient with HSC-BPPV after CRP treatment.

In supine roll tests of patients with HSC-BPPV, horizontal nystagmus was elicited by placing the head in the right and left positions (positional nystagmus), as well as during the process of head-turning (head-turning nystagmus). In general, the direction of nystagmus was the same as that of head turning. Further, the intensity of head-turning nystagmus was weaker and that of positional nystagmus stronger. After a latency period, positional nystagmus elicited by the right/left position first rose to peak intensity, then weakened and gradually disappeared. Duration ranged from 6 to 81 s (mean 27.17 s) on the affected side, and 6–74 s (mean 22.76 s) in the unaffected side. Positional nystagmus was caused by canalolithiasis and manifested as bilateral geotropic horizontal nystagmus, accompanied by a weak vertical upward nystagmus, which was more pronounced on the affected side, and disappeared following CRP treatment (Figure 1). Head-turning nystagmus was elicited by the process of head-turning from supine to the lateral position (head-left and head-right) and vice versa in the supine roll test, both before and after CRP treatment (Figure 1). Head-turning nystagmus was also detected in the healthy subjects, and characterized by horizontal nystagmus in the same direction as head turning during the supine roll test, consistent with the findings in patients with HSC-BPPV; however, no positional nystagmus was detected in healthy subjects.

### Head-Turning Nystagmus on Supine Roll Test of Patients With HSC-BPPV and Healthy Subjects

**Figure 2:**
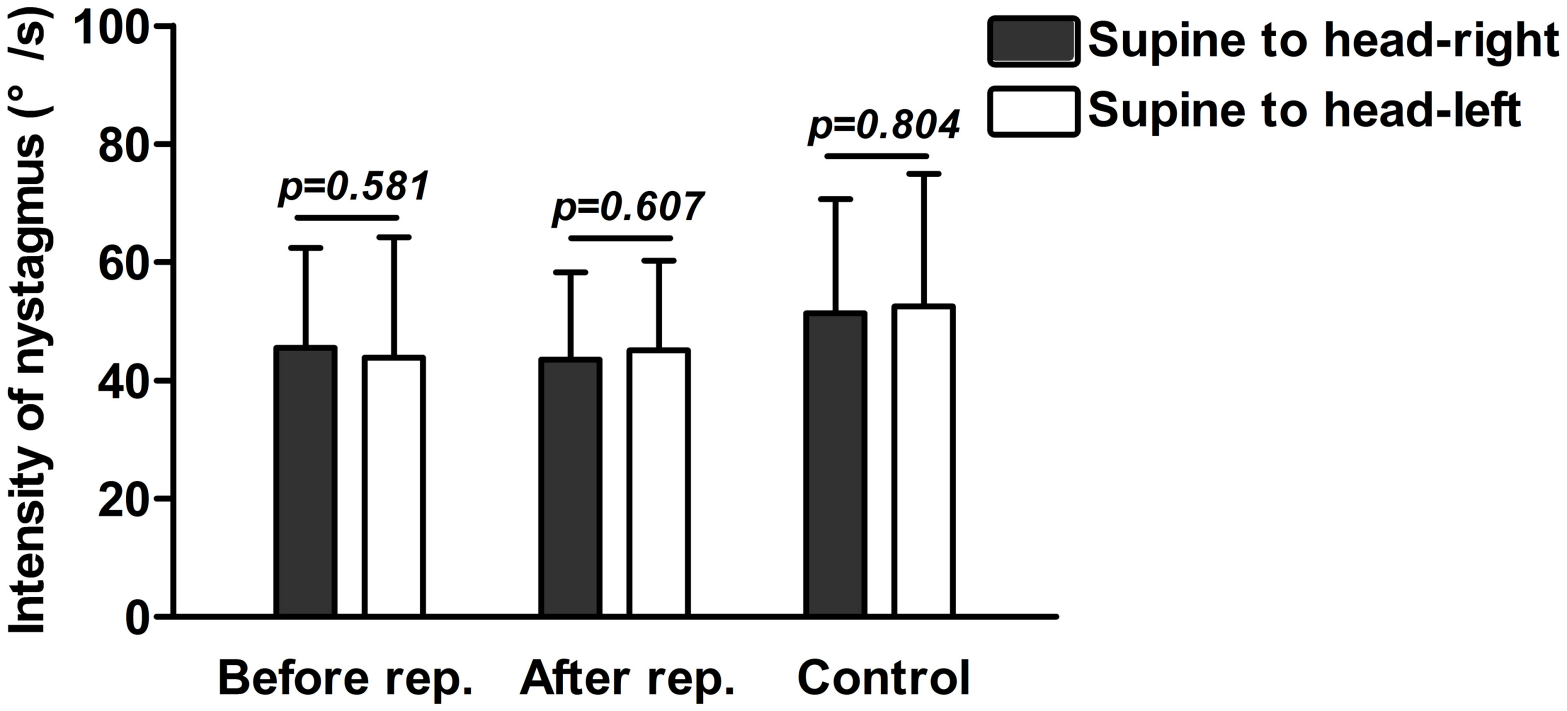
Head-turning nystagmus on supine roll test of patients with HSC-BPPV with/without CRP treatment and healthy subjects. **Before rep**.: Head-turning nystagmus on supine roll test of patients with HSC-BPPV without CRP treatment. **After rep.:** Head-turning nystagmus on supine roll test of patients of HSC-BPPV with CRP treatment. **Control:** Head-turning nystagmus on supine roll test of healthy subjects.

The ranges of head-turning nystagmus intensity were 11.5–88.9°/s (mean 45.5°/s) and 11.6–98.5°/s (mean 43.8°/s) elicited by turning from the supine to head-right and head-left positions, respectively, in the 72 patients with HSC-BPPV. After CRP treatment, intensity ranged from 20.7 to 84.6°/s (mean 43.5°/s) and 20.8–86.3°/s (mean 45.1°/s) in 49 patients. We also analyzed head-turning nystagmus elicited by head-turning from supine to the affected and unaffected sides in patients with HSC-BPPV; no difference was detected between the two groups either before or after CRP treatment (*p* = 0.987 and *p* = 0.488, respectively). Intensity ranged from 21.9 to 100°/s (mean 51.3°/s) and 16.3–101.3°/s (mean 52.5°/s) in the 38 healthy subjects. In summary, there was no significant difference in head-turning nystagmus of HSC-BPPV patients, either before or after repositioning, or in healthy subjects (*p* > 0.05) (Figure 2).

### Positional Nystagmus on Supine Roll Test of Patients With HSC-BPPV

**Figure 3:**
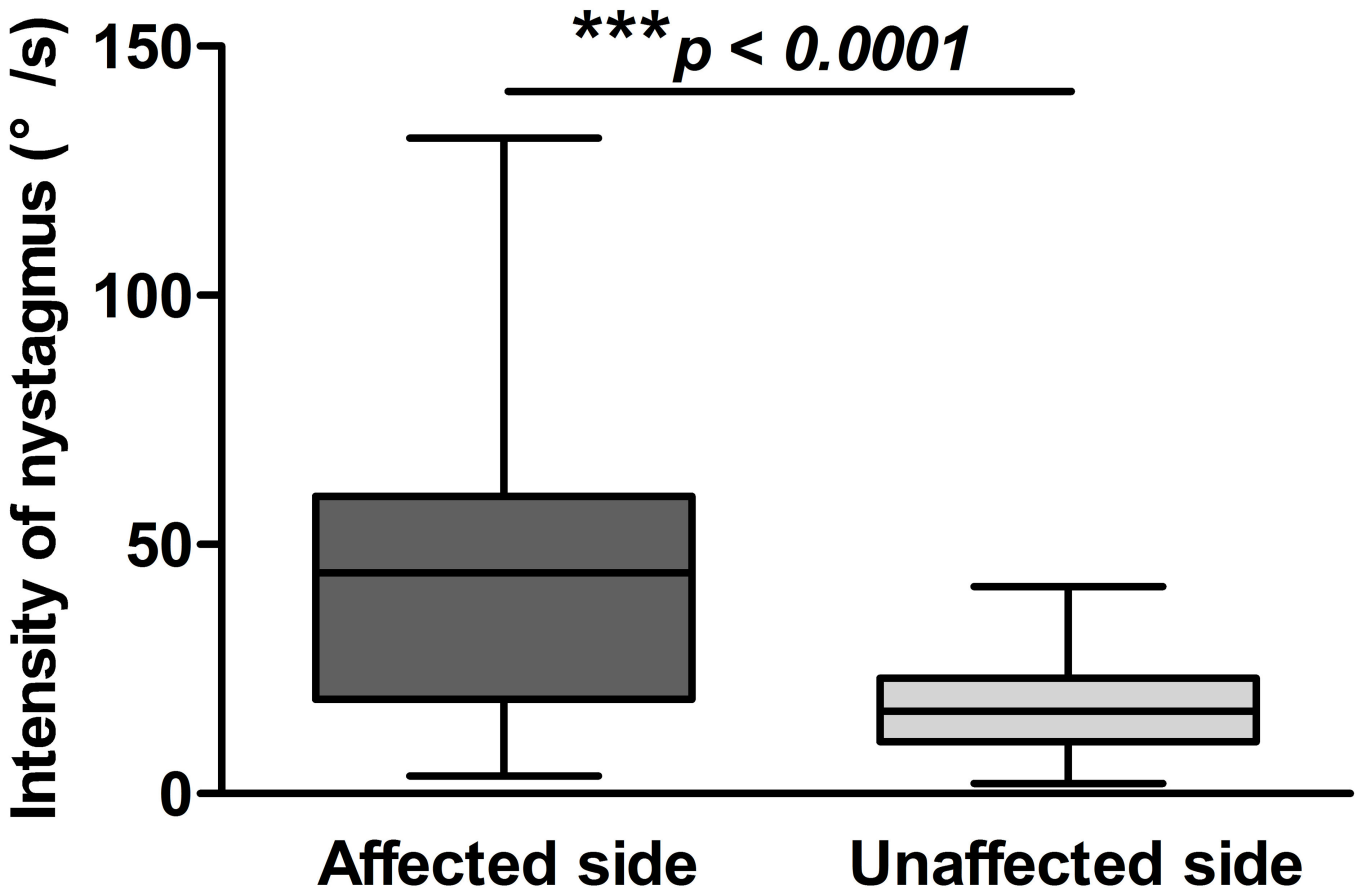
Positional nystagmus on the affected and unaffected sides in 72 patients with HSC-BPPV. Positional nystagmus elicited on the affected and unaffected sides on supine roll test of patients with HSC-BPPV. The intensity values of positional nystagmus differed significantly between the affected and unaffected sides, with values of 40.81 ± 25.56°/s (range, 3.5–131.6°/s) and 17.69 ± 9.31°/s (range, 2.1–41.6°/s), respectively, and a ratio of (2.59 ± 1.98):1.

The latency, duration, and intensity of positional nystagmus on supine roll test were recorded and analyzed for 72 patients with HSC-BPPV. On lying with the affected ear down, horizontal geotropic nystagmus was observed, with a mean onset latency of 1.78 ± 2.11 s (range, 0–10 s), and the mean duration of positional nystagmus was 27.17 ± 13.78 s (range, 6–81 s). Rolling patients onto the unaffected side resulted in markedly lower values for both latency and duration, at 1.06 ± 1.72 s (range, 0–10 s) and 22.76 ± 11.89 s (range, 6–74 s), respectively (*P* < 0.05). The characteristics of positional nystagmus in 72 patients with HSC-BPPV are presented in Table 2. Further, the intensity values of positional nystagmus on the affected and unaffected sides were 40.81 ± 25.56°/s (range, 3.5–131.6°/s) and 17.69 ± 9.31°/s (range, 2.1–41.6°/s), with a ratio of (2.59 ± 1.98):1, representing a significant difference between groups (*P* < 0.0001) (Figure 3).

**Table 2:** Characteristics of positional nystagmus in 72 patients with HSC-BPPV.

**Head position**	**Latency (s)**	**Duration (s)**	**Intensity (°/s)**
Affected side	1.78 ± 2.11	27.17 ± 13.78	40.81 ± 25.56
Unaffected side	1.06 ± 1.72	22.76 ± 11.89	17.69 ± 9.31
*t-*value	2.238	2.055	7.209
*p-*value	0.027	0.042	0.000

Next, we compared head-turning and position nystagmus on the affected and unaffected sides in 72 patients with HSC-BPPV before CRP treatment. Positional nystagmus was markedly more intense on the affected side than that on the unaffected side (*p* < 0.0001); however, head-turning nystagmus intensity did not differ significantly between the two sides (*p* = 0.987). In particular, positional nystagmus was markedly less intense than head-turning nystagmus (*p* < 0.0001) when the patient was moved from the supine position to the unaffected side, while there was no difference between the two groups when patients were moved from supine to the affected side (*p* = 0.294) (Table 3).

**Table 3:** Comparisons of nystagmus on the affected and unaffected sides in 72 patients with HSC-BPPV.

	**Head-turning nystagmus (°/s)**	**Positional nystagmus (°/s)**	***t-*value**	***p-*value**
Affected side	44.70 ± 18.24	40.81 ± 25.56	1.053	0.294
Unaffected side	44.65 ± 19.27	17.69 ± 9.31	10.690	0.000
*t-*value	0.016	7.209		
*p-*value	0.987	0.000		

## Discussion

Benign paroxysmal positional vertigo is common, sometimes terrifying, but rarely portends serious disease. It is usually easily diagnosed and treated, and both the patient and the physician are immediately gratified. While much has been learned about the pathogenesis of BPPV in the past decades, many of its features remain mysterious (11). Previously, the physiological characteristics of BPPV were studied through biomechanical models (12, 13) and animal models (14). However, a functional model of a single semicircular canal has not been established in human body. According to Flourens' and Ewald's law, we know that the rotation plane of nystagmus is consistent with the plane of excited semicircular canal, but the excitatory or inhibitory effects have not been quantified.

Here, we focused on establishing a functional model of a single semicircular canal and chose HSC-BPPV for several reasons. In patients with HSC-BPPV, the horizontal nystagmus, rather than the torsional vertical nystagmus, can be elicited by a change in head position relative to gravity. HSC-BPPV was resulted from displacement of an otoconial mass within the endolymph of the HSC. Otoconia roll in opposite directions, due to gravity, when the head is turned to the affected and unaffected sides, stimulating the HSC of the affected side with the same factor bilaterally, producing a physiological stronger excitatory effect and weaker inhibitory effect, respectively. However, stimulation by the otoconia had yet to begin during the process of head-turning in the supine roll test, while physiological head-turning caused the endolymph to flow in the opposite direction of head-turning. Horizontal semicircular canals on both sides were stimulated simultaneously, eliciting horizontal nystagmus with the same direction as the head turning and no difference in intensity.

According to Ewald's law (15), rolling of the otoconia from the posterior arm of the HSC toward the ampulla in the affected side due to gravity drives the endolymph to the ampulla (as angular acceleration stimulation is applied to the semicircular canal alone) in the supine roll test, which excites the hair cells and elicits horizontal nystagmus, accompanied by weak vertical nystagmus. When the head is turned to the unaffected side, the otoconia rolls to the canal from the ampulla, driving the endolymph away from the ampulla (as angular deceleration stimulation is applied to the semicircular canal alone), inhibiting the hair cells in the affected side and eliciting a weaker horizontal nystagmus than the one triggered by the excitatory stimulus (16, 17). Further, the process of head-turning from the supine to the lateral position (head-left and head-right) and vice versa in the supine roll test is similar to that in the rotational test, which can apply angular acceleration and deceleration stimuli on bilateral horizontal semicircular canals, respectively, resulting in horizontal nystagmus in the same direction as head turning. A weak vertical nystagmus can be induced in the supine roll test because of the anatomic structure of the HSC in the inner ear. Della Santina et al. (18) reported that the mean horizontal semicircular canals plane tilted slightly up laterally 20° above Reid's horizontal planes, which was defined as a plane passing through the center of each bony external auditory canal (at the lateral entrance of the tympanic bone) and the cephalic edge of the inferiormost aspect of each infraorbital rim. According to Ewald's law, we know that the rotation plane of nystagmus is consistent with the plane of excited semicircular canal. Hence, geotropic horizontal nystagmus, accompanied by a weak vertical upward nystagmus, was caused by canalolithiasis. Based on the factors leading to BPPV (19) (i.e., otoconia loss, head displacement, and semicircular canal well) and the interaction between otoconia and endolymph in a semicircular canal, HSC-BPPV could be regard as a show of Ewald's law in human body theoretically.

At present, research into the function of the HSC is mainly conducted using caloric and rotational tests, because of the specific anatomical structure involved and the limitations of screening equipment. However, it is difficult to stimulate the unilateral horizontal semicircular canal with a single factor using either approach. Inhibitory or excitatory effects on the HSC can be caused by a single temperature (cold or warm) stimulus, and the dual effects of positive and negative stimuli cannot be determined simultaneously. Alternate warm and cold stimulation of both ears can reflect the dual effects of positive and negative stimuli; however, it is challenging to ensure that both effects are equal (5). Therefore, the caloric test can only predict that the bilateral horizontal semicircular canal should have an equal effect (nystagmus) in response to equal stimuli, but has not been standardized and is not quantitative. The lesion side, according to the relative difference in bilateral intensity of nystagmus, is evaluated first. The rotational test can be used to evaluate the function of the HSC by rotating around the vertical axis, which simulates physiological horizontal rotation stimuli; however, the horizontal semicircular canals on both sides receive inhibitory or excitatory stimuli simultaneously during this test. Therefore, this approach can only reflect comprehensive effects on both sides, despite only using a single stimulus on one side. That is, the rotation test cannot reveal physiological effects on a single horizontal semicircular canal receiving positive and negative stimuli. There have been ongoing efforts in the study of vestibular medicine to establish a functional model of a single semicircular canal and assess its effects according to Ewald's laws, which can facilitate exploration of its physiological properties.

In this study, we analyzed the characteristics of nystagmus on supine roll test in 72 patients with HSC-BPPV, 49 of whom were treated with CRP, as well as 38 healthy subjects, and found that head-turning nystagmus was in the same direction as head turning in all groups and that its intensity did not differ significantly among groups. Positional nystagmus was also elicited in patients with HSC-BPPV, but not healthy subjects. Nystagmus features, including latency, duration, and intensity, differed significantly between the affected and unaffected sides. These results suggest that there are physiological and pathological differences in the nystagmus induced by the supine roll test, corresponding to head-turning and positional nystagmus. The head-turning nystagmus was elicited during head turning from supine to the lateral position and vice versa in both patients with HSC-BPPV with/without CRP treatment and healthy subjects. The positional nystagmus, elicited at the right/left position after a latency, resulting from the displacement of an otoconia by gravity, can also be regarded as physiological nystagmus. The intensity values of positional nystagmus elicited on the affected and unaffected sides of the head were 40.81 ± 25.56°/s and 17.69 ± 9.31°/s, respectively, with a ratio of (2.59 ± 1.98):1. Ichijo (20) reported that the intensity of nystagmus elicited on the affected and unaffected sides of the head varied markedly among 20 patients with HSC-BPPV, with a ratio of 5:2, which is broadly consistent with our findings. Further, we showed that there was no positional nystagmus on supine roll test of patients with HSC-BPPV treated with CRP, due to the absence of otoconia stimulus, while head-turning nystagmus was still elicited, and did not differ significantly from that observed in healthy subjects. Notably, although there was no significant difference, the intensity of head-turning nystagmus in patients with HSC-BPPV with or without CRP treatment was weaker than that in healthy subjects, possibly due to semicircular canal dysfunction in these patients. The lesion of semicircular canals has the same etiological factors with the utricle pathological change in BPPV, and the dysfunction mostly happens in low-frequency range of semicircular canal frequency band. The ectopic otoconia is not the main etiological factors for that (21). The ectopic otoconia would be returned back to utricle after CRP treatment, but the dysfunctional of the semicircular canals remained. Therefore, the intensity of nystagmus in patients with BPPV after CRP treatment was weaker than that in healthy subjects. Our findings suggest that supine roll testing of patients with HSC-BPPV can both reflect the direction and intensity of the nystagmus elicited by equal excitation and inhibition on a single horizontal semicircular canal, and clarify the gradient of those effects, which will be helpful in understanding the functional status of single horizontal semicircular canals.

## Conclusion

HSC-BPPV is considered a clinical disease; however, the nystagmus, elicited by the displacement of otoconia and semicircular canals on supine roll test follows the laws of Flourens and Ewald. This is reflected in the direction and intensity of nystagmus elicited by a single horizontal semicircular canal, indicating that it has physiological properties. These finding suggest that HSC-BPPV can be a show of Ewald's law in human body, and used as a physiological stimulus model to understand deeply the characteristics of the human horizontal semicircular canal.

## Data Availability Statement

The raw data supporting the conclusions of this article will be made available by the authors, without undue reservation.

## Ethics Statement

The studies involving human participants were reviewed and approved by The Ethics Committee of the Tianjin First Central Hospital. The patients/participants provided their written informed consent to participate in this study.

## Author Contributions

WW and TC: study design. TC, WW, SL, QL, CW, and XZ: acquired and analyzed the data. XZ, YB, WW, XH, and TC: drafted the manuscript. All authors data interpretation and critical revision of the manuscript.

## Conflict of Interest

The authors declare that the research was conducted in the absence of any commercial or financial relationships that could be construed as a potential conflict of interest.
